# Determining factors for the increase in self-referrals to the Emergency Department of a rural hospital in Huelva (Spain)

**DOI:** 10.1371/journal.pone.0207199

**Published:** 2018-11-28

**Authors:** Enrique Pino-Moya, Mónica Ortega-Moreno, Juan Gómez-Salgado, Carlos Ruiz-Frutos

**Affiliations:** 1 Intensive Medicine and Emergencies CMU, Riotinto Hospital, Huelva,Spain; 2 Economy Department, University of Huelva, Huelva,Spain; 3 Nursing Department, University of Huelva, Huelva, Spain; 4 Safety and Health Posgrade Program, Universidad Espíritu Santo, Guayaquil, Ecuador; 5 Preventive Medicine and Public Health, University of Huelva, Huelva, Spain; University of Mississippi Medical Center, UNITED STATES

## Abstract

**Objective:**

To analyse the increase of self-referral patients at the Emergency Department of Riotinto District Hospital (in Huelva, Spain) during a short period. The study focused on patients’ profiles to identify key factors that explained the increase of self-referrals.

**Material and methods:**

Retrospective descriptive study using patient’s data from a hospital emergency department between 2003–2015, excluding the period 2012–14 due to the lack of records. Socio-demographic variables, type of referral, access to health services, hospital route, transfer time and organisational changes were analysed, among other factors. Descriptive statistics, chi-square test, and binary logistic regression analysis were used.

**Results:**

Self-referral patients to the hospital emergency department revealed a growing trend. Logistic regression model showed that the variables that best predict its occurrence were the health system changes from 2008 and the time it takes to get to the Extra-hospital Emergency Services, where those changes act as modifiers of the effect. From 2008, the likelihood of self-referral in towns with an Extra-hospital Emergency Service over 2 minutes away by car was of 76.43%. When including the triage level, the logistic regression model showed that 83.1% of patients referred themselves.

**Conclusions:**

Changes in the health system and in the time for patients to get to the reference hospital from their origin, affect the likelihood of self-referral to the emergency department. Once the patient's severity level was included, this variable, along with the time to get to the emergency department, modified the probability of self-referral to the emergency department. We found an increase in hospital services together with a reduction of resources in the primary care emergency system. This may have led to inefficiencies in the public health system, together with an increase in self-referrals and greater problems to service users.

## Introduction

Patients who attend the Emergency Department (ED) by their own initiative, without being referred by any health professional or institution, are known as self-referral patients [1]. According to the literature, this is one of the causes for the improper use of services [2] which results in ED overcrowding [3]. Factors influencing self-referral include socio-cultural and organisational changes [4], age, sex, socioeconomic status, accessibility to primary care, doctor-patient relationship, geographical area, and health condition or severity of the episode [5]. As Brezzi et al. stated, self-referral is increasing in developed countries and is attributed to different causes such as lack of accessibility or lack of trust in other levels of care, perceived severity, etc. [6].

**Fig 1:**
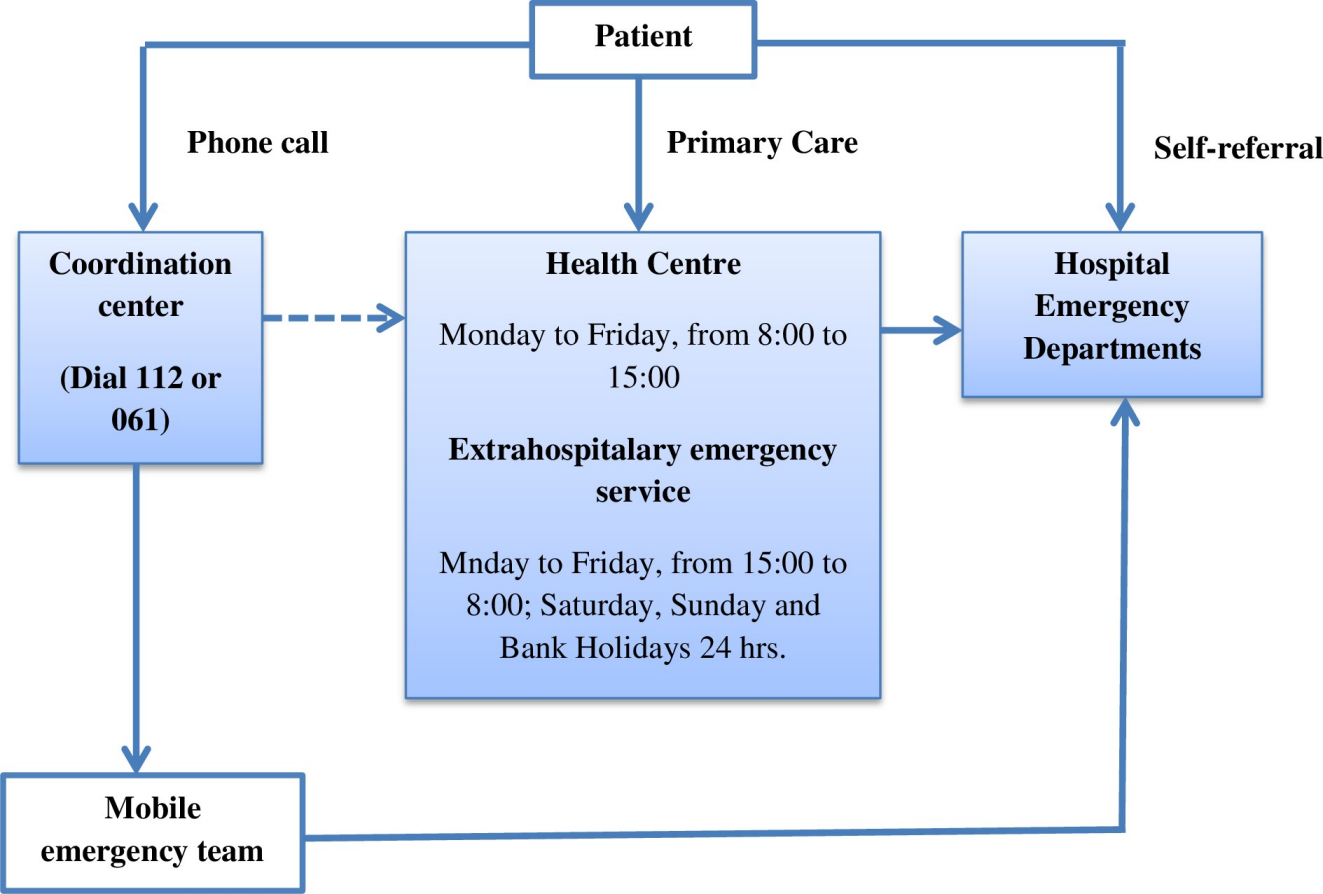
The Spanish National Health System levels of care provision to respond to emergency situations.

The Spanish National Health System is built on public founding, full coverage, free and equal accessibility and almost full benefits. It has three levels of care provision to respond to emergency situations: primary health care centres, Extra-hospital Emergency Services (EHES) and hospital ED, and a coordination centre that responds to telephone consultations. Individuals have different ways to request and receive health care in emergency situations. Fig 1 shows a diagram of these routes. In Spain, self-referral is the most frequent form of attendance to ED, representing 75.4% of the cases [7].

**Fig 2:**
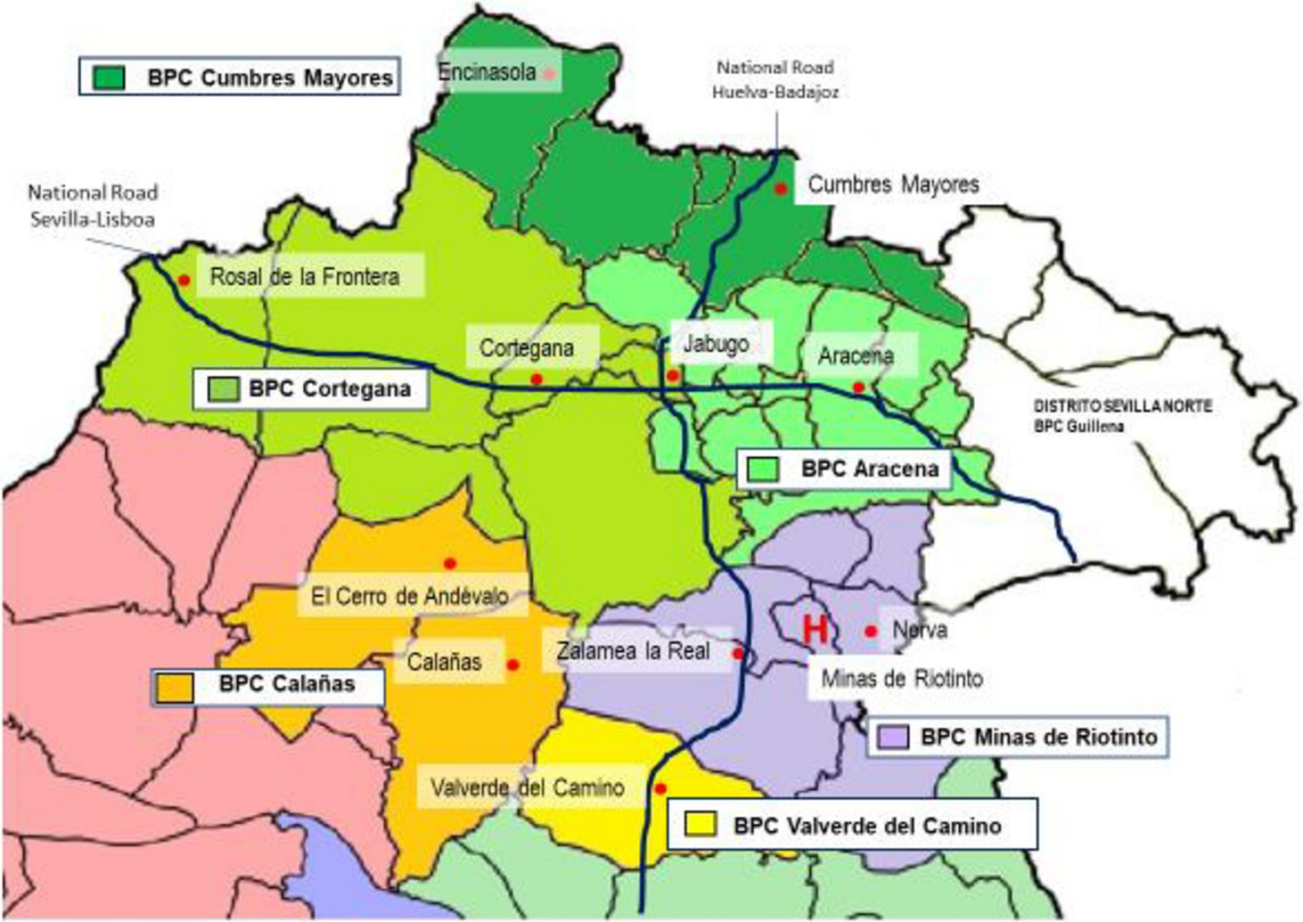
Location of the Basic Primary Care Areas, Extra-hospital Emergency Services and Riotinto Hospital.

The Northern Huelva Health Area is a predominantly rural and remote region, with over 50% of the population living in rural municipalities of less than 10,000 inhabitants. The area is mostly mountainous, has 3,707 km^2^ and a population of 69,921 inhabitants. The population density is low (18.6 inhabitants / km^2^). As a consequence, communications are difficult, which means that more than half of the population needs more than 45 minutes to reach the nearest city [7]. Northern Huelva Health Area has six basic primary care areas and 11 EHES with mobile equipment for urgent care. Specialised assistance, including urgent and critical care, is provided at the Riotinto Hospital [7]. Fig 2 shows the location of the different resources of the Northern Huelva Health Area.

The increase of self-referral patients in the Riotinto Hospital ED, from 24.28% (5261) in 2003 up to 70.20% (17912) in 2011 and 62.63% (16915) in 2015, led us to investigate the factors that were involved in these changes.

As a hospital located in a rural area, as described above, ED receives patients referred predominantly from primary care centres. However, during the period of this study, self-referrals reached similar percentages similar to national figures.

During the period referred in this study, the organisational and functional structure of Northern Huelva Health Area had an unequal distribution of quotas for primary care level. The general practitioner/health centre ratio was 1.02, with only one doctor in 9 out of the 11 EHES and in the 18 health centres. These doctors were responsible for a high demand work of around 1,450 emergencies per 1,000 inhabitants in the EHES. There was also a shortage of paediatricians in the health centres, which affected their normal activity. Furthermore, critical care transport equipment was not available in the area.

Between 2006 and 2009, there was a public employment and transfer tender that involved the dismissal of 29 of the 61 medical general practitioners. From the structural point of view, there were two important events: cessation of the afternoon activity in three health centres (2009), and closing of the Minas de Riotinto EHES, without an increase in the resources of those centres to cope with the demand (2008).

### Aim of the study

The aim of this study is to assess the possible factors that have contributed to the increase of self-referral patients at Riotinto Hospital Emergency Department.

## Material and methods

### Design and sample

This study followed a retrospective observational design. A total of 242,475 patients that referred themselves to the Riotinto Hospital ED were included. Out of them, 215,639 patients were assisted between January 2003 and December 2011, and 26,869 patients were assisted during 2015. Although it was recorded, the data from 2012 to 2014 was excluded due to an error on the registration programme and its codification. During this period, item “self-referral” was renamed as “transport by own means”, including every patient who was not transferred by an arranged ambulance, even in those cases where they were referred by a general practitioner. Patients were recorded and classified according to the mean of transport instead of type of referral. So, the research team decided to exclude information from 2012–2014 as it could confound results and conclusions. In 2015, the item “transport by own means” was renamed “self-referral” again.

The manuscript has been checked using the STROBE Statement for observational studies.

### Variables

Socio-demographic variables (sex, age, origin) were analysed, as well as type of referral (self-referral or referred), the hospital route, transfer time (higher or lower than the median value), availability of the EHES in town, access to daily general practitioners (GP) consultation, triage (only in data form 2015), destination (admission or discharge) and period (due to organisational changes occurred in 2008). Regarding the hospital route, there was a difference between the patients who lived in a town with EHES (or on their way to the hospital), and patients who lived in a location with the same distance from EHES and from the hospital.

### Instruments and analysis

Descriptive study by distribution of frequencies and percentages. The chi-square test, along with the odd ratios (OR) and associated confidence intervals, and a logistic regression analysis were calculated to identify those variables that played an important role. As well as to build an assessment model for the type of referral (self-referral or referred), identifying the possible modifying and/or confounding effects of the variables. Coefficients were estimated by maximum likelihood, with forward selection. Measures for goodness of fit verified the model as appropriate. Hosmer-Lemeshow test percentage of correctly classified values, sensitivity, and specificity. The model with most precision of effects and most quality advantages in the final variables, importance, and simplicity was chosen.

Due to a significant period during which data was excluded (because of the codifying error), the data from 2003–2011 was analysed and reported separately from the 2015 data. IBM statistical software SPSS Statistics 20.0 was used. Data was obtained from the Hospital Admission *Aurora* and *DIRAYA-Emergencies* software, regarding the Northern Huelva Health Area. Transfer time and distance between the different locations and the out-of-hospital emergency services or the hospital, were estimated with Google Maps taking into consideration the shortest route.

### Ethics approval and consent to participate

The data was provided by the Area Department of Statistics and, both this and its processing, stuck to the guidelines of the Organic Law on Protection of Personal Data (15/1999, December 13).

## Results

The total number of patients assisted at the Riotinto Hospital ED between the years 2003 and 2011 and in the year 2015 was 242,508, out of which 125,446 (51.7%) attended on self-referral.

Women recorded more frequent visits to the ED (51.5%), and the mean age of patients assisted was 43.69 years (SD “standard deviation” 26.85), being the largest group those between 15 and 65 years of age. 66.5% of patients came from basic health areas non-adjacent to Riotinto Hospital. 52.2% of them did not have EHES in their town, 4.2% did not have access to daily GP consultations, and in 25.5% of the cases the hospital route was shorter than the EHES route. Out of the total patients treated, 84.3% were discharged directly from the area of consultation.

The mean time of access to the EHES was 6.88 minutes (SD 6.55), taking less than 2 minutes for 50% of the patients. Regarding the transfer time to the hospital, the mean transfer time was 28.44 minutes (SD 19.55); for 50% of the patients, it took a maximum of 27 minutes.

Self-referral patients attending the ED increased from 5% between 2003–07 to 62.2% in 2008, 70.4% in 2011, and 62.9% in 2015. The increase of self-referral contrasted with the decrease of referrals from EHES, which decreased from more than 50% to below 20%. Referrals from general practitioners (GP) were constant (10.6 ± 2.85%).

From 2003 to 2011, 215,639 patients were treated in the hospital emergency department and 50.3% of them were self-referral. **Table 1** shows statistical significant differences between self-referral patients versus patients referred by a health professional. Not having a daily GP, consultation is at the limit of the OR = 1.044 (1–1.090). In addition, the self-referral is higher among those who are eventually discharged versus those requiring hospitalisation and further care from 2008 onwards (after organisational changes mentioned above), presenting a risk 3.861 times higher than that of the patients previously assisted (OR = 3.861 (3.793–3.931)).

**Table 1:** Bivariate analysis, years 2003–2011.

	TOTAL		DECISION							
			Self-referral		Referred					
	N	%	N	%	N	%	*x*^2^	P-value	OR	95% IC for OR
Total	215,639	100%	108,531	50.3%	107,108	49.7%				
**Sex**										
0. *Male	105,056	48.7%	51,522	49.0%	53,534	51.0%	135.404	<0.001	1.106	1.087–1.124
Female	110,535	51.3%	56,980	51.5%	53,555	48.5%				
**Age**										
0. 65 years or more (Elder people)	62,637	29.0%	25,716	41.1%	36,921	58.9%	3,037.43	<0.001	1.694	1.662–1.726
< 65 years	153,002	71.0%	82,815	54.1%	70,187	45.9%				
0. < 15 and ≥ 65 years	100,712	46.7%	46,550	46.2%	54,162	53.8%	1,276.28	<0.001	1.362	1.339–1.385
Between 15 and 64 years (adults)	114,927	53.3%	61,981	53.9%	52,946	46.1%				
0. ≥ 15 years	177,568	82.3%	87,699	49.4%	89,869	50.6%	356.24	<0.001	1.238	1.211–1.266
From 0 to 14 years (children)	38,071	17.7%	20,832	54.7%	17,239	45.3%				
**Basic Health Area**										
0. Non-adjacent	143,665	66.6%	66,403	46.2%	77,262	53.8%	2,907.38	<0.001	1.642	1.613–1.672
Adjacent	71,974	33.4%	42,128	58.5%	29,846	41.5%				
**Extrahospital Emergency Service—Riotinto Hospital Emergency Department**										
0. Not returning	160,863	74.6%	74,908	46.6%	85,955	53.4%	3,588.25	<0.001	1.824	1.788–1.860
Returning	54,776	25.4%	33,623	61.4%	21,153	38.6%				
**Extrahospital Emergency Service in town**										
0. YES	88,785	44.1%	37,865	42.6%	50,920	57.4%	2,641.76	<0.001	1.590	1.563–1.619
NO	112,381	55.9%	60,894	54.2%	51,487	45.8%				
**Daily consultation**										
0. YES	192,435	95.7%	94,383	49.0%	98,052	51.0%	3.85	0.05	1.044	1–1.090
1. NO	8,731	4.3%	4,376	50.1%	4,355	49.9%				
**Time to Extrahospital Emergency Service**										
0. ≤ 2 minutes	114,762	57.0%	50,100	43.7%	64,662	56.3%	3,161.23	<0.001	1.664	1.635–1.694
> 2 minutes	86,404	43.0%	48,659	56.3%	37,745	43.7%				
**Time to Riotinto Hospital Emergency Department**										
0. > 27 minutes	97,159	48.3%	42,573	43.8%	54,586	56.2%	2,092.63	<0.001	1.506	1.480–1.533
≤27 minutes	104,007	51.7%	56,186	54.0%	47,821	46.0%				
**Destination**										
0. Admission	31,610	14.7%	14,696	46.5%	16,914	53.5%	210.33	<0.001	1.193	1.165–1.222
Discharge	183,537	85.3%	93,434	50.9%	90,103	49.1%				
**Period**										
0. 2003–2007	112,568	52.2%	39,152	34.8%	73,416	65.2%	22,777.21	<0.001	3.861	3.793–3.931
2008–2011	103,071	47.8%	69,379	67.3%	33,692	32.7%				

The total of cases per variable does not always correspond with the total of patients, as there is no data record for some patients. *Value “0” or “1” before the modality of each variable indicates the codification used for the analysis, assigning value “1” to the category exposed to risk and “0” to the basal or reference category.

The probability of attending the emergency department by self-referral from 2003 to 2011 from the binary regression model follows the following formula:

$$P(Self-referral)=\frac{1}{{1+e}^{-(-0.891+0.455T\_EHES+1.325Period+0.129T\_EHESxPeriod))}}$$

where T_EHES indicated whether the time from the town of origin to the EHES of reference is lower, equal or greater than the median; and *Period* assessed if the patient attended before or after 2008.

The period had a modifying effect, homogeneity test with a 44.47 chi-square statistic (p-value<0.001). The Wald test assessed the individual statistical significance of each of the estimated coefficients (p-value<0.001 in all cases), and the omnibus test, with the statistic of the likelihood ratio LR = 25393.902 (p-value<0.001), allowed to affirm that the variables included in the model effectively contributed to explaining the modifications that took place in the probability of attending ED on the patients’ self-referral. The Hosmer and Lemeshow test did not reject the logistic regression model (p-value = 1). The selected model obtained a sensitivity of 62.8% and a specificity of 69.2%, correctly classifying 66% of the patients. The probability of attending on self-referral depending on the period and whether the town is more than 2 minutes away from the EHES is collected in **Table 2**.

**Table 2:** Estimated values of the logistic regression model. 2003–2011.

Time to the Extra-hospital Emergency Service	Period	Self-referral Probability
Less than or equal to 2 minutes	Attends between 2003 and 2007	29.09%
Less than or equal to 2 minutes	Attends between 2008 and 2011	60.68%
More than 2 minutes	Attends between 2003 and 2007	39.27%
More than 2 minutes	Attends between 2008 and 2011	73.46%

Regarding analysis from data obtained in 2015, 26,869 patients were assisted at the ED, 63% of them on self-referral. As a novelty, it was possible to analyse the severity levels classified by the Spanish System of Triage, consolidated that same year. Among the patients with lower severity (triage 4 and 5) the self-referral predominated, 10,646 cases (70%). However, this percentage decreased to 53.6% (6,254 patients) in the highest levels of severity (triage 1, 2, and 3).

The bivariate contrasts made to study the association between different factors and the fact of attending ED on self-referral or referral in the year 2015 were significant. **Table 3** collects the statistics of the chi-square test, odd ratios and confidence intervals, together with the information relative to the total frequencies and by category.

**Table 3:** Bivariate analysis. Year 2015.

	TOTAL		DECISION							
			Self-referral		Referral					
	N	%	N	%	N	%	*x*^2^	P-value	OR	95% CI for OR
	26,869	100%	16,915	63.0%	9,954	37.0%				
**Sex**										
0. *Male	12,476	46.4%	7,581	60.8%	4,895	39.2%	47.93	<0.001	1.192	1.134–1.252
Female	14,386	53.6%	9,330	64.9%	5,056	35.1%				
**Age**										
0. 65 years or more (Elder people)	7,742	28.8%	3,773	48.7%	3,969	51.3%	942.88	<0.001	2.310	2.188–2.438
< 65 years	19,127	71.2%	13,142	68.7%	5,985	31.3%				
0. < 15 y ≥ 65 years	12,419	46.2%	7,058	56.8%	5,361	43.2%	371.02	<0.001	1.630	1.551–1.713
Between 15 and 64 years (adults)	14,450	53.8%	9,857	68.2%	4,593	31.8%				
0. ≥ 15 years	22,192	82.6%	13,630	61.4%	8,562	38.6%	128.82	<0.001	1.482	1.385–1.587
From 0 to 14 years (children)	4,677	17.4%	3,285	70.2%	1,392	29.8%				
**Basic Health Area**										
0. Non-adjacent	17,640	65.7%	10,113	57.3%	7,527	42.7%	696.41	<0.001	2.086	1.974–2.204
Adjacent	9,229	34.3%	6,802	73.7%	2,427	26.3%				
**Route Extra-hospital Emergency Department-Riotinto Hospital Emergency Department**										
0. Not returning	19,876	74.0%	11,541	58.1%	83,350	41.9%	782.56	<0.001	2.397	2.252–2.551
Returning	69,93	26.0%	5,374	76.8%	1,619	23.2%				
**Extra-hospital Emergency Services in town**										
0. YES	10,017	41.4%	5,366	53.6%	4,651	46.4%	460.05	<0.001	1.775	1.684–1.871
NO	14,189	58.6%	9,533	67.2%	4,656	32.8%				
**Daily consultation**										
0. YES	22,720	93.9%	13,933	61.3%	8,787	38.7%	7.99	0.005	1.172	1.050–1.308
NO	1,486	6.1%	966	65.0%	520	35.0%				
**Time to Extra-hospital Emergency Services**										
0. ≤ 2 minutes	13,338	55.1%	7,315	54.8%	6,023	45.2%	564.77	<0.001	1.901	1.803–2.006
> 2 minutes	10,868	44.9%	7,584	69.8%	3,284	30.2%				
**Transfer time**										
0. >27 minutes	11,072	45.7%	6,031	54.5%	5,041	45.5%	432.23	<0.001	1.738	1.649–1.831
≤ 27 minutes	13,134	54.3%	8,868	67.5%	4,266	32.5%				
**Destination**										
0. Admission	5,656	21.2%	2,916	51.8%	2,710	48.2%	380.96	<0.001	1.803	1.698–1.913
Discharge	20,931	78.8%	13,811	66.0%	712	34.0%				
Priority**										
0. Triage 1.2 o 3	11,659	43.4%	6,254	53.6%	5,405	46.4%	756.26	<0.001	2.016	1.917–2.120
Triage 4 o 5	15,210	56.6%	10,646	70.0%	4,564	30.0%				

The total of cases per variable does not always correspond with the total of patients, as there is no data record for some patients.

*Value “0” or “1” before the modality of each variable indicates the codification used for the analysis, assigning value “1” to the category exposed to risk and “0” to the basal or reference category.

**The priority distinguishes the severity level or triage performed with the Spanish Triage System (STS).

To determine if the new variable was confounding in the model, a bivariate analysis was carried out, stratified according to the priority of the patient and obtaining a higher risk (OR = 2.143) for those less serious patients (triage 4 and 5).

The homogeneity test between the OR values of the two strata determined the existing differences, with the statistic 9.489 (p-value = 0.002), and the interaction was considered when studying the model.

The selected model in 2015 can be summarised in the equation:

$$P\;(Self-referral)=\frac{1}{{1+e}^{-(-0.136+0.601T\_EHES+0.614PRIORITY+0.161T\_EHESxPRIORITY)}}$$

where PRIORITY indicates the level of triage or severity.

For the proposed model, the Wald test assessed the individual statistical significance of the coefficients (p-value<0.001 in all cases). The variables in the model contributed to explain the probability changes for a patient to attend the hospital ED on his/her self-referral as the omnibus test is rejected, with statistic 1467.676 (p-value<0.001). The model adjustment and the Hosmer and Lemeshow test were accepted (p-value = 1). Finally, the proposed model correctly classified 64.4% of the patients, with a sensitivity of 32.8% and a specificity of 83.1%. **Table 4** collected the probabilities associated with the model, depending on the time to arrive to the EHES and the patient classification by the Spanish Triage System.

**Table 4:** Estimated values of the logistic regression model. 2015.

Time to the Extra-hospital Emergency Service	Priority	Self-referral Probability
Less than or equal to 2 minutes	Triage 1.2 o 3	46.61%
Less than or equal to 2 minutes	Triage 4 o 5	61.73%
More than 2 minutes	Triage 1.2 o 3	61.42%
More than 2 minutes	Triage 4 o 5	77.56%

## Discussion

The analysis of self-referral increase in the Riotinto Hospital ED has helped us to understand better the profile and patterns of patients who attend it, and thus, to identify the factors that would allow us to plan health resources more efficiently, showing a very similar behaviour–in particular regarding self-referral-, to the described in the literature (age, sex, distance, time, etc.) [8,9,10,11].

As previously mentioned, in the years excluded from the study, from 2012 to 2014, the figures of self-referral were excessively high (80–85%). This could be caused by a misinterpretation in the assignment of the origin of the patients, an error in the coding when recording the data. For this reason, it was decided not to include them since they could distort the results with a "false positive".

The increase of self-referrals seems to be due to the loss of accessibility to health resources [12,13]. This is explained by structural causes, inherent to the socio-demographic context and the dispersion of the Northern Huelva Health Area which, with its mountainous geography, causes longer transfer times between towns and health resources. This affected negatively the timeliness of emergency services response and the distribution of human resources, which together with the absence of specific critical care transport equipment, affected the activity of the EHES in those places with a single doctor, forcing their closure, and increasing self-referrals.

Geographic accessibility and EHES availability have been regarded as facilitating variables. In fact, the absence of EHES in the local area was influential when deciding whether to attend the hospital on a self-referral basis, as well as the route the patient had to perform to receive emergency care. Patients whose EHES was in the opposite direction to that of the hospital or in an alternative route, were more likely to attend the Riotinto hospital ED on self-referral. Furthermore, when quantifying the time, it was found that self-referrals at the hospital were favoured because of the proximity to the centre (EHES) due to greater transfer times from the town to the EHES. This outcome coincides with previous studies that found that a larger distance to their primary health care centre is associated with an increased attendance to the hospital ED [14,15]. In addition, people who lived closer to the hospital [16,17] and in urban areas [18,19] made a greater use of the hospital ED and self-referral. Even so, in the period after 2008–2015, 55% of those living more than 50 minutes away referred themselves; twice the percentage than that of 2003–2007.

From the organisational point of view, we need to consider the possible influence produced by the cessation of afternoon activity in some health centres and the closing of the “Minas de Riotinto” EHES, since this has an impact on the demand to the health area and the hospital ED. It has been described that the increase in the use of EHES does not reduce hospital emergencies [20], that the use of health services is seen globally, and there is no replacement of primary care and hospital health care levels [21]. However, in our study, we found a decrease of these extrahospital resources. The number of patients who attended the Riotinto Hospital Emergency Department, who belonged to the area EHES, had a 20% increase between the years 2008 and 2011, from 7500 to 9000 patients, with a peak in 2009 of 10500 patients. Of these patients, 80% attended on self-referral. The number of patients coming from other EHES showed very stable figures and, although there was a general increase in self-referral, this was lower (50%-60%).

Similarly, another factor that could have had an influence is the reduction in number of physicians, which was caused by the long periods of no transfer possibilities, and the modifications of their form of recruitment. This affected the physician-patient relationship. Changes were also made in the assignment of contracts; in the doctors’ replacements, where no fixed assignment of quotas was made and with a decrease in the number of replacements regarding the days (-29,9%) and the number of contracts (-47,2%) [22,23].

At the same time, the introduction of improvements and innovations in the hospital service portfolio could increase the confidence and expectations regarding the ED [24]. Note that for patients who lived far from the hospital or in rural populations, the quality of the service was the major factor that influenced the decision making over attending the Riotinto hospital [25]. To this we must add the low accessibility to specialised attention (barriers between different organisation levels) [26].

The adjustment of our data to a binary logistic regression model emphasised the fact that the changes produced in the health system in 2008 and the time to get to the emergency department simultaneously affected the probability of attending on self-referral, where the first element acts as an effect modifier factor. Once the structural and organisational changes were saved and a quantifiable variable of the patient's priority was introduced, in the year 2015, the level of severity or triage and the time to get to the EHES were the factors that influenced the probability of attending the ED on the patients’ self-referral. At the same time, forcing those patients with more severity levels (triage 1–3) and with a greater distance to the EHES to attend the ED on their self-referral, with greater probability than those who live near their EHES (61.4% vs 46.6%); this probability is similar to that of patients with lower severity levels (triage 4–5) and who live closer to the EHES (61.7%).

Apart from analysing each variable of the health system and its repercussion in the increase of self-referral to the Riotinto Hospital ED, we assessed the set of modifications that have enhanced the Hospital's Health Services portfolio with the creation of an ICU, day hospital and palliative care services, pacemaker implantation service, etc., and those modifications that have reduced the Primary Care Emergency System services, with the cessation of activity in some health centres, the closing of the Minas de Riotinto EHES and the loss of the referring physician as examples of these effects. The increase in self-referrals to the hospital ED is a sign of inefficiency of the system that generates greater health expenses and service difficulties, due to the need of transfer to this ED and the subsequent care delay. However, there is another vision through which the user gets a higher quality of health care that compensates for such inefficiency.

## Conclusions

This work has allowed us to describe factors regarding the demand for emergency care of a specific population, with particular socio-demographic characteristics, population dispersion, resources, and a health care system that influences the behaviour of patients attending the Riotinto Hospital ED. We have seen in this study that the probability of self-referring to ED is influenced by the changes in the health system and the time it takes for the patient to get from their town to the EHES. Once the patient's severity level was introduced, it was this variable, along with the time to get to the emergency department, which modified the probability of self-referring to the emergency department. Secondly, it allowed us to use trend analysis to better anticipate future population behaviour, if the conditions remain unchanged.

The changes in the organisation and health care must be studied from the point of view of its impact, not only in terms of morbidity and mortality, but also taking into account the patients’ behaviour when using resources and the accessibility that these resources have. Thus, the studied population, rural and remote, changed the pattern of origin of the patients who come to the hospital ED from being predominantly referred from primary care to self-referred [8]. These findings coincide with previous studies in which the lower Index Multiple Deprivation (IMD) is the main predictive factor for the use of emergency services [27].

The great population dispersion, represented by the time of access to the health services, is a determining element that has acted as a necessary factor to expand and maintain the effects of the changes produced in the health system of the Northern Huelva Health Area.

### Study limitations

The reasons for consultation and the final diagnosis could not be analysed due to their lack of coding. Diagnosis codes might help tackle this issue in the study. Patients may often be unable to judge the severity of their condition and may have considered non-urgent symptoms as urgent. Health problems are the main motivation for patients to attend the ED on self-referral, even for patients with non-urgent symptoms, and a health professional can refer these patients to either a GP or the ED [28]. In addition, the level of triage of the Aurora software could not be obtained, and in the Diraya-Emergencies a variation was identified in the model used. The year’s range “2012–2014” was discarded because of changes in the origin consignment items in the Emergency Admissions program, substituting the “self-referral” item for "transportation by own means" thus including all except those derived by ambulance. Another limitation was the absence of some demographic data not found in the records: marital status, employment situation, level of education, socioeconomic status, and ethnic background.

## Supporting information

S1 Table STROBE Checklist. (DOCX)
